# Draft Genome Sequence of *Bacillus altitudinis* YNP4-TSU, Isolated from Yellowstone National Park

**DOI:** 10.1128/genomeA.00631-17

**Published:** 2017-07-13

**Authors:** Joshua A. OHair, Hui Li, Santosh Thapa, Matthew Scholz, Suping Zhou

**Affiliations:** a Department of Agricultural and Environmental Sciences, Tennessee State University, Nashville, Tennessee, USA; b Vanderbilt Technologies for Advanced Genetics (VANTAGE), Vanderbilt University Medical Center, Nashville, Tennessee, USA

## Abstract

Undisturbed hot springs inside Yellowstone National Park remain a dynamic biome for novel cellulolytic thermophiles. We report here the draft genome sequence of one of these isolates, *Bacillus altitudinis* YNP4-TSU.

## GENOME ANNOUNCEMENT

A new strain, *Bacillus altitudinis* YNP4-TSU, was isolated from Whiterock Springs (lat 44.780233, long 110.69805), which is inside Yellowstone National Park, USA. The rather new species *B. altitudinis* was first discovered in 2006 from cryogenic tubes taken at 41 km in the atmosphere (1). Since then, only six other *B. altitudinis* strains, including YNP4-TSU, have been deposited in the NCBI genome database (retrieved May 12, 2017). This species has the ability to produce spores that can withstand some of the most extreme environments, ranging from atmospheric radiation (1) to geothermal heated springs. 

*B. altitudinis* YNP4-TSU was isolated by vacuum filtration in 0.22 µm Millipore systems from water samples of 59°C and a pH of 2.3. Filters containing unknown amounts of specimens were then cut and transferred to nutrient agar (2). Areas with substantial growth were then re-streaked and incubated at 37°C to produce individual colonies. *B. altitudinis* YNP4-TSU tested positive for extracellular endoglucanase activity on 10% carboxymethylcellulose (CMC) under the Congo red assay (3). After positive cellulase testing, whole genomic DNA was extracted using the GenElute Sigma Genomic DNA kit for Gram-positive strains (Sigma, USA) (3). For genome sequencing, libraries were prepared with Illumina TruSeq DNA Nano sample kits using indexed adaptors (Illumina). Pooled libraries were subjected to 150-bp paired-end sequencing according to the manufacturer’s protocol (Illumina HiSeq3000). Bcl2fastq2 conversion software (Illumina) was used to generate demultiplexed Fastq files. This work was performed at the Vanderbilt Technologies for Advanced Genomics (VANTAGE) at Vanderbilt University (Nashville, TN, USA). Raw reads were then trimmed to remove bases of *Q* average ≤ 3 using Burrows–Wheeler alignment (4). *De novo* assembly was performed using SPAdes version 3.7.1 (5) with default parameters and the “-careful” flag. The draft genome of YNP4-TSU was assembled into 67 contigs with a total genome size of 3,749,504 bp (*N*_50_, 172,320) and a G+C content of 45.0%. Automated annotation was performed with the NCBI Prokaryotic Genome Annotation Pipeline (PGAP) version 3.3 and yielded 3,734 coding genes, 72 tRNAs, and 16 rRNAs. Annotation predicted several endoglucanase, exoglucanase, and cellobiase genes, which upon an NCBI nucleotide BLAST search (retrieved April 25, 2017) revealed many novel gene-encoding sequences (6). These potential enzymes may have an important role on future biomass fermentation, which is why a further examination of the enzymatic rates will help determine the cellulolytic capabilities of *B. altitudinis* YNP4-TSU.

### Accession number(s).

The whole-genome shotgun project reported here has been deposited at DDBJ/EMBL/GenBank under the accession number MEDE00000000. The version reported here is the first version, MEDE01000000.
